# Field evaluation of pyriproxyfen and spinosad mixture for the control of insecticide resistant *Aedes aegypti *in Martinique (French West Indies)

**DOI:** 10.1186/1756-3305-3-88

**Published:** 2010-09-16

**Authors:** Frédéric Darriet, Sébastien Marcombe, Manuel Etienne, André Yébakima, Philip Agnew, Marie-Michelle Yp-Tcha, Vincent Corbel

**Affiliations:** 1 Institut de Recherche pour le Développement (IRD), Laboratoire de Lutte contre les Insectes Nuisibles, 911 Avenue Agropolis, BP 64501, 34394 Montpellier Cedex 5, France; 2 Centre de démoustication, Conseil Général de la Martinique, BP 679 Avenue Pasteur, 97200 Fort de France, Martinique; 3 Génétique et évolution des maladies infectieuses, Unité mixte de recherche 2724, Centre National de la Recherche Scientifique, Institut de Recherche pour le Développement, 911 Avenue Agropolis, BP 64501, 34394 Montpellier Cedex 5, France; 4 Institut de Recherche pour le Développement, Centre de Recherche Entomologique de Cotonou, 01 BP 4414, Cotonou, Bénin

## Abstract

**Background:**

The resistance of *Ae. aegypti *to insecticides is already widespread and continues to develop. It represents a serious problem for programmes aimed at the control and prevention of dengue in tropical countries. In the light of this problem measures to control *Ae. aegypti *are being orientated towards how best to use existing insecticides, notably by combining those that have different modes of action.

**Results:**

In this study we evaluated the operational efficiency of a mixture composed of pyriproxyfen (an insect growth regulator) and spinosad (a biopesticide) against a population of *Ae. aegypti *from Martinique resistant to pyrethroid and organophosphate insecticides. The first step consisted of evaluating the efficacy of pyriproxyfen and spinosad when used alone, or in combination, against *Ae. aegypti *larvae under simulated conditions. The results showed that the mixture of pyriproxyfen+spinosad remained active for at least 8 months, compared with 3 months for spinosad alone, and 5 months for pyriproxyfen alone. In a second step in containers experiencing natural conditions, pyriproxyfen and spinosad, maintained the rate of adult emergence at 20% for 3 weeks and 3.5 months, respectively. Following the same criteria of evaluation, the mixture pyriproxyfen+spinosad remained effective for 4.5 months, showing that the combination of the two larvicides with different modes of action acted to increase the residual activity of the treatment.

**Conclusion:**

The mixture of pyriproxyfen and spinosad kills larvae and pupae giving it a broader range of action than either insecticide. This mixture could preserve the utility of both insecticides in public health programs.

## Background

*Aedes aegypti *(L.) is the principle vector of dengue worldwide, causing 50-100 million cases of infection and 30,000 deaths each year [1]. There is no specific medication or vaccine available to deal with the arbovirus responsible and the only means of controlling the disease is to control its mosquito vectors. The first line of vector control is to physically eliminate breeding sites where water collects. However, it is not possible to eliminate all sites and those that remain need to be treated with efficient and long-lasting insecticides active against larvae and/or pupae. Mosquito control agencies are constrained by the range of insecticides available to them and would like a broader range of products that could be used. However the development of new families of chemicals acting against novel targets is rare, requiring years of research in the laboratory and field. In addition resistance to organophosphates, carbamates and pyrethroids in mosquitoes is on the rise and includes many populations of *Ae. aegypti *[2-7]. Pyriproxyfen (a growth regulator) and spinosad (a biopesticide) have recently been evaluated for their action against mosquitoes. Field and laboratory studies have found pyriproxyfen (active against the pupal stage) to have good residual activity against *Ae. aegypti *[8]. Spinosad (active against larvae) has been found to have low toxicity for humans and other non-target fauna [9] and has the potential to be used against mosquitoes as it does not show cross-resistance with conventional insecticides [10]. Research into new strategies aimed at limiting the development of insecticide resistance in mosquitoes has been orientated towards the use of mixtures of two insecticides, where each one possesses a different mode of action. In the laboratory, the association of pyriproxyfen and spinosad has been found to be synergistic in that it increases the mortality of *Ae. aegypti *larvae, thereby also reducing the number of emerging adults [11].

In this study we investigated the performance of a mixture of pyriproxyfen and spinosad in field conditions on the island of Martinique in the French West Indies against a population of *Ae. aegypti *resistant to both pyrethroids and organophosphates [7]. The two insecticides used separately or when mixed together were evaluated in containers protected from the weather and those experiencing natural conditions present in the community of Vauclin in the south-east of the island. The main aim was to test the relative efficacy of the pyriproxyfen and spinosad mixture against that of the two insecticides when applied individually in two settings calling for minimal or maximal dosages to be used.

## Materials and methods

### Mosquito material

The strain of *Ae. aegypti *used in this study was collected in the community of Vauclin, Martinique (14°54'N, 60°84'W) and larvae of the F_1 _progeny were used for the simulated trials. This population showed strong resistance to pyrethroid and organophosphate insecticides due to the presence of a *Kdr *mutation [f(R) = 0.71; V1016I] and increased metabolic detoxification (*i.e *oxidases, esterases, glutathione S-transferases) [7].

### Insecticides

Pyriproxyfen is a juvenile hormone analogue that is particularly active against pupal stages of development. It disrupts insect hormonal regulation and results in the inhibition of development, disturbed behaviour, and an important decrease in adult fertility [12]. It has low toxicity for mammals with an LD_50 _above 5000 mg/kg for rats [13]. Spinosad is an insecticide of natural origin composed of a mixture of two metabolites (spinosins A and D) produced by the soil bacterium *Saccharopolyspora spinosa *(Actinomycetes). Its mode of action is unique as it acts against both GABA and nicotinic receptors in the insect nervous system [14]. It has low toxicity for mammals with an LD_50 _of 3783 - 5000 mg/kg for rats [13].

### Simulated field trial

**Figure 1:**
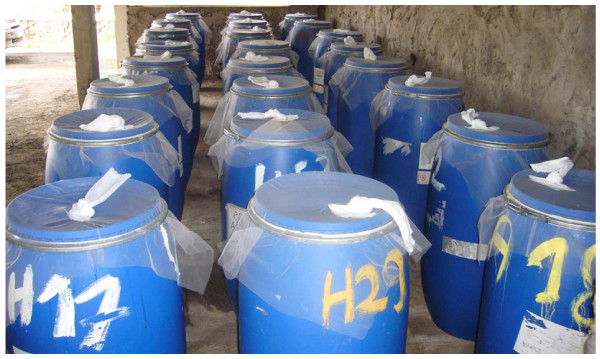
**Plastic 175 litre containers used in the trial of larvicides against *Ae. aegypti *in simulated field trial**.

A phase II trial was conducted at Fort-de-France (Martinique) following the standard procedures of the World Health Organisation (WHO) [15]. The efficacy and residual activity of pyriproxyfen [Sumilarv^®^, granules (GR) 0.5%] and of spinosad [granules (GR) 0.5%] used alone or in combination were measured in plastic containers of a type that represents the most productive larval breeding sites of *Ae. aegypti *in Martinique [16]. The 175 litre containers were filled with 145 litres of tap water and then covered with mosquito nets held in place by a metal clamp to prevent oviposition by wild female mosquitoes from the area (Figure 1). These containers were placed undercover where they were protected from the sun and rain. The trial involved a total of 12 containers, where 3 were treated with pyriproxyfen (0.02 mg/l), 3 with spinosad (0.1 mg/l), 3 with a mixture of pyriproxyfen and spinosad (0.02 mg/l + 0.1 mg/l), and 3 were untreated and served as controls. The doses of 0.02 mg/l of pyriproxyfen and 0.1 mg/l of spinosad represent the minimal doses recommended by the WHO for the control of *Ae. aegypti *larvae [17,18]. At the moment of treatment, and every 10 days thereafter for the rest of the trial, 100 third instar *Ae. aegypti *larvae of the Vauclin strain (F_1 _progeny) were added to each container and their survival was followed until adult emergence. Each time larvae were added to a container, the container was refilled with water to maintain a constant volume against water lost due to evaporation (~200-300 ml every 10 days) and 1 g of ground cat food biscuits was added to provide food for the larvae.

### Trials in natural breeding sites

**Figure 2:**
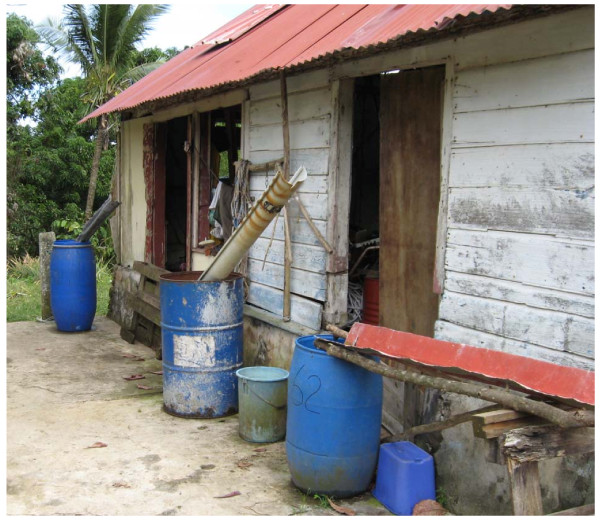
**Containers of *Ae. aegypti *exposed to natural variation from the site "Cadette" used in the study**. The three largest containers were treated.

The trials were conducted from February to August 2008 in the community of Vauclin, situated in the south-east of Martinique on the Atlantic coast. Three isolated sites were chosen at least 2 km from one another, to avoid possible migration of mosquitoes between locations; Anse Maroquet (14°33'N, 60°49'W), Château Paille (14°33'N, 60°50'W) and Cadette (14°33'N, 60°52'W). Anse Maroquet is a fishing village situated in a bay, Château Paille is a housing development, and Cadette a collection of houses situated approximately 4 km further inland. This region of the island has a humid tropical climate with rainy season running from May to September with an annual precipitation of ~2000 mm. In each locality, 20 containers (100-200 litres capacity) positive in *Ae. aegypti *larvae were chosen for the trials, i.e., 5 containers for each of the 3 insecticide treatments and 5 for the untreated control (Figure 2). The quantity of insecticide added to containers was calculated as a function of their holding capacity without taking into account the volume of water present; this was not controlled and varied during the course of the trial. The formulation of pyriproxyfen used was the same as that used in the simulated trials involving the protected containers (GR 0.5%). For spinosad, the formulation used was in the form of a direct application tablet (DT 7.48%). The concentration of 0.05 mg/l of pyriproxyfen and the equivalent of 1 spinosad tablet for 200 litres of water (= 0.5 mg/l) represent the maximum doses recommended by the WHO [17,18]. The concentration of the mixture involved a total of 0.05 mg/l of pyriproxyfen and 0.5 mg/l of spinosad. The allocation of treatments to containers within each site was done at random, with 5 containers receiving the same treatment in each site. The density of larvae and pupae in each container was evaluated prior to treatment and 2 days following treatment (D_0 _and D_2_), and then once a week thereafter (D_7_, D_14_, D_21_, D_28_, D_35_,...). The sampling method was based on 3 dips with a small fish net and by counting the number of 3rd and 4th instar larvae, as well as the number of pupae. First, 2nd and 3rd instar larvae were replaced in their respective containers, while 4th instar larvae and pupae were brought back to the laboratory in 200 ml of water from the same container to estimate adult emergence. Each treated container was followed in the field until the relative density of larvae was > 20% of its initial density (D_0 _= 100%) and the percentage emergence of adults was ≥ 20% (equivalent to ≤ 80% inhibition of emergence) [15]. The sampling of some containers was abandoned during the course of the trial in cases where they were completely emptied (e.g. due to domestic use) or disappeared.

### Statistical analyses

The data collected from the trials in the artificial and natural containers were analysed separately using a split-plot design for the analyses of variance (ANOVA) with repeated measures for the different times of sampling (see below for details).

In the simulated field trial, the time (in days) required for the inhibition of adult emergence (*%IE*) to pass below the threshold value of 80% was estimated for each treatment according to the formula [15]: $\%IE=\frac{C-T}{C}\times 100$ where *C *represents the average percentage emergence in the untreated (control) containers at a particular time and *T *represents the average percentage emergence in the treated containers in the same period.

For the trial involving containers in natural conditions, calculations were based on the relative density (RD) of 3rd and 4th instar larvae and pupae in each container, for each location, day of sampling and insecticide treatment. This analysis did not directly test variation in the density of control larvae, but data from control containers were incorporated in the estimation of RD according to the following equation: $\%RD=\left(\frac{C1}{T1}\right)\left(\frac{T2}{C2}\right)\times 100$ where *C1 *is the average number of 3rd/4th instar larvae and pupae in the 5 control (untreated) containers in a particular site at time D_0_; *T1 *is the average number of 3rd/4th instar larvae and pupae in the 5 treated containers of a particular treatment and site at time D_0_; *C2 *is the average number of 3rd/4th instar larvae and pupae in the 5 control containers of a particular site and day of sampling, and *T2 *is the number of 3rd/4th instar larvae and pupae in each treated container of the insecticide treatments, per site and day of sampling. Relative densities were analysed until D_140_, as after that date, the number of containers yielding data was insufficient for analysis. In the laboratory, percentage adult emergence was calculated using the formula: $\%E=100-\left(\frac{C-T}{C}\right)\times 100$ where *C *and *T *were as above for the containers in protected conditions. A split-plot ANOVA was used to analyse the inhibition of emergence (*%IE*) and relative density (*%RD*) of mosquitoes in containers exposed to natural variation. The whole plot level of the design involved the three locations (treated as a nominal random effect), and the three insecticide treatments (treated as a nominal fixed effect). The error term for the whole plot involved the interaction between the factors location and treatment. The model also included a term for container within location and treatment to account for random variation among replicate containers. The sub-plot level of the model involved the factor 'day of sampling' as a nominal fixed effect and the interaction between day and treatment; these were tested against the remaining residual error variation.

Percentage data were logit transformed (log [%*x*/(1 - %*x*)]) for analyses to standard errors within the bounds of 0 - 100% when back-transformed to the percentage scale. In the analysis of %RD, the estimates of average values (e.g. the average number of control larvae in replicate containers from a particular site at time zero) were calculated on the scale of log (*x *+ 1) to help stabilise estimates and back-transformed. Calculations leading to negative estimates of %RD were set to zero. The percentage data were scaled to values between 0 and 1 using the formula; 0.05 + 0.99*x *prior to logit transformation. Models were analysed using the Restricted Maximum Likelihood method (REML) of JMP^® ^version 7.0.1 [19].

## Results

### Simulated field trials

**Figure 3:**
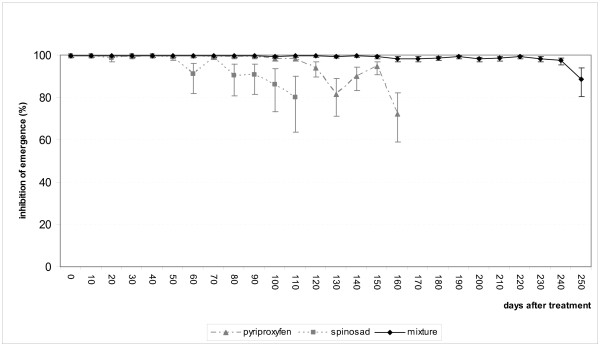
**Simulated field trial**. Residual activity of pyriproxyfen and spinosad when used alone or as a mixture on the inhibition of emergence for *Ae. aegypti *(± s.e.).

The results of this trial for the containers treated with insecticides are presented in Figure 3. The rate of emergence inhibition for adult *Ae. aegypti *fell below the threshold value of 80% after 110 days (~3.5 months) for spinosad and after 160 days (~5.3 months) for pyriproxyfen. Following the same criteria, the activity of the mixture of pyriproxyfen+spinosad lasted for at least 250 days (~8 months), that is, 1.6 - 2.0 times longer than for spinosad and pyriproxyfen when each was used alone. Mortality in the control containers (not shown) generally did not go above 10%, except in sampling periods 1 (10 days), 5 (50 days) and 11 (110 days) where mortality was > 20%.

### Trials in natural breeding sites

**Figure 4:**
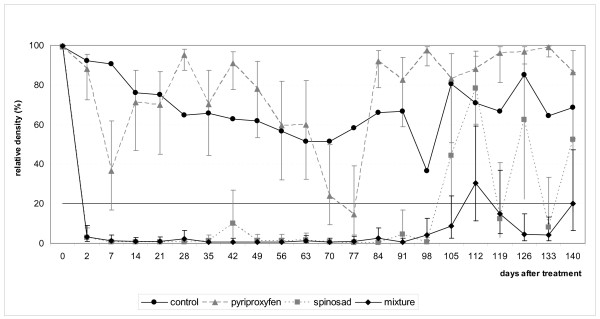
**Trial in natural breeding sites**. Relative density (RD) of third and fourth instars larvae and pupae of *Ae. aegypti *before and after treating containers with pyriproxyfen, spinosad and their mixture (± s.e.).

**Figure 5:**
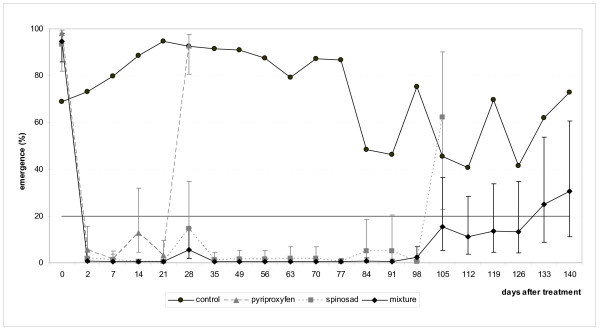
**Trial in natural breeding sites**. Percentage adult emergence of *Ae. aegypti *adults in the laboratory before and after treatment of containers with pyriproxyfen, spinosad and their mixture (± s.e.).

The factor location (Anse Maroquet, Château Paille, Cadette) and its interaction with different treatments had little influence on the results of these trials as the coefficients of variance accounted for <1% and ~5% of variance explained by the ANOVA models for relative density and inhibition of emergence (Table 1). A greater proportion of the variation in the data could be attributed to variation among containers within a particular treatment and location (Table 1). The density of larvae/pupae in the control containers varied over the course of the trial, decreasing relative to its initial value and then increasing again after ~70 days (Figure 4). Calculations for the relative density of larvae/pupae in the treated containers take this variation into account. There was no consistent pattern in the relative density of larvae/pupae exposed to pyriproxyfen which fluctuated considerable over the course of the trial (Figure 4). However it is difficult to estimate the efficiency of this insecticide in the field based on larval counts as many of the observed larvae, though still alive, would not have completed their development to the adult stage. For spinosad, the relative density of pre-adults passed the 20% threshold and regained 45% of their initial numbers 105 days after treatment (~3.5 months). With the mixture of pyriproxyfen+spinosad, the relative density of pre-adults rebounded to 30% of its initial value after 112 days, but then fell again to below 20% in the subsequent samples until D_140 _(~4.5 months)(Figure 4). Data from larvae and pupae brought back to the laboratory allowed calculation of the 20% threshold for adult emergence (or 80% inhibition of emergence) beyond which it is recommended to treat again. This threshold was reached after 21 days for pyriproxyfen, 105 days for spinosad, and 133 days for the mixture of pyriproxyfen+spinosad (Figure 5). It was interesting to note the dramatic drop in the efficacy of pyriproxyfen between days 21-28. We cannot currently explain this observation but think it merits attention in future studies. In the containers treated with spinosad and the mixture of the two larvicides, it was interesting to note that the time needed for pre-adult populations to recover 20% of their initial size coincided with the time necessary for adult emergence to reach 20% in laboratory conditions.

**Table 1:** Split-plot repeated measures analysis of variance for treatment effects on *Ae. aegypti *in the field experiments.

Source	N	DFnum	DFden	F	*P*	CV
*Relative Density*						
Treatment (T)	3	2	3.9	9.8	0.03	-
Day (D)	21	20	516.8	10.8	< 0.001	-
DxT	110	84	516.5	0.9	0.5	-
Locality (L)	3	-	-	-	-	< 1%
LxT	15	-	-	-	-	4.61%
Container (C) [LxT]	45	-	-	-	-	7.3%
						
*Emergence inhibition*						
Treatment (T)	3	2	503	16.8	< 0.001	-
Day (D)	22	21	503	12.8	< 0.001	-
DxT	66	42	503	1.5	0.03	-
Locality (L)	3	-	-	-	-	< 1%
LxT	9	-	-	-	-	5.3%
Container (C) [LxT]	45	-	-	-	-	24%

N = number of parameters,

DFnum = degrees of freedom numerator,

DFden = degrees of freedom denominator,

CV = coefficient of variance.

## Discussion

Ecological considerations are increasingly important and strategies of mosquito control must give priority to insecticides that are effective and respect the environment. Correspondingly, a tightening of European directives (Directive Biocide 98/8), has tended to exclude compounds from the families of organochlorides, organophosphates and carbamates from use in public health programs. Mosquito control agencies in Europe now often have no other alternative than to use the biopesticide *Bacillus thuringiensis *var *israelensis (Bti) *[20]. If suspected cases of resistance to *Bti *in *Culex pipiens *from New York [21] are excluded, there are no cases of mosquito resistance to *Bti *as the different toxins in its parasporal crystals act in synergy [22]. Resistance to spinosad has not been reported for mosquitoes. The Vauclin strain of *Ae. aegypti*, which is already resistant to pyrethroid and organophosphate insecticides, shows a decreased susceptibility to pyriproxyfen with a significant resistance ratio (RR_50 _= 2) relative to the susceptible Bora strain [23]. This difference in susceptibility could be explained by the different genetic backgrounds of the two strains and/or due to the over expression of the numerous detoxifying enzymes responsible for the pyrethroids and organophosphates resistance, hence conferring a "cross tolerance" to pyriproxyfen. In Brazil it has already been found that some populations of *Ae. aegypti *resistant to temephos have cross-resistance to a juvenile hormone analogue, methoprene [24]. Insecticides cause strong selection pressures on their target populations, and their prolonged use often leads to the evolution of resistance to the compound concerned and those with the same or similar modes of action. The use of a mixture composed of two insecticides with different modes of action acting on different targets diminishes the short-term risk that resistance will arise to one or the other of the active compounds [25]. In the case of the association between pyriproxyfen and spinosad, the efficacy of the first against the pupal stage combines with that of the second, which acts more specifically against larvae. This complementary action translates into a powerful synergistic effect at concentrations close to the LC_99 _[11].

In the containers protected from the sun and weather, the mixture of pyriproxyfen+spinosad remained active for at least 8 months against the Vauclin strain of *Ae. aegypti *in comparison to 3.5 months and 5 months for spinosad and pyriproxyfen, respectively, when applied alone. When the treated waters were in containers protected from sunlight and bad weather, the two larvicides alone and when mixed were remarkably stable. In contrast in the containers exposed to natural variation, pyriproxyfen was only effective for 21 days. Following the same criteria, spinosad remained active for 3.5 months and the mixture for 4.5 months. At doses comprised between 0.02 and 0.05 mg/l, the WHO estimate 1 month of activity for pyriproxyfen [17]. With a residual activity of 3.5 months in the natural containers, spinosad (= 0.5 mg/l) was shown to be a promising candidate for the control of *Ae. aegypti*. The WHO estimates its activity to be 2 - 3 times less at dose of 0.1 and 0.5 mg/l [18]. The mixture of pyriproxyfen+spinosad had advantages that neither of the two insecticides had when used alone. In particular the rapid action of spinosad on larvae complements the action of pyriproxyfen and leads to the rapid mortality of both larvae and pupae in a site. This provides a strong practical advantage as the effects of treatment can be rapidly assessed by health workers directly in the field. In contrast, treatments involving insect growth regulators, such as ecdysteroids (diflubenzuron, triflumuron, novaluron, teflubenzuron, etc...) or juvenile hormones (methoprene, fenoxycarb, pyriproxyfen), take longer to act and do not kill all the individuals present in a site. This is not to say such treatments are not efficient in reducing adult numbers. What it does mean, however, is that laboratory bioassays are often required to verify if any remaining larvae or pupae will contribute to the adult population or not. This will necessarily add to the costs of control in terms of both time and money, compared with treatments where both larvae and pupae are rapidly killed.

Insecticides that work in synergy when mixed together are an avenue to explore in mosquito control for the needs of public health. Negative aspects of such combinations are those shared with conventional insecticides, in that resistance is ultimately expected to evolve in response to prolonged use and that it is not possible to clearly predict how efficient mixtures will remain if resistance to one of the compounds already exists or develops. Nonetheless, combinations of insecticides with different modes of action could make an efficient contribution in the fight against mosquitoes, notably in regions where mosquitoes already show high levels of resistance to conventional insecticides. The availability of new families of insecticides has been scarce in the last 10 years and relying on the appearance of new products is not a realistic option for the control of resistant populations in the short- to medium-term future. In contrast, the option of associating insecticides with different modes of action is available now. This is a concept currently too often overlooked in public health, although in agricultural practice mixtures of insecticides have been used for more than 20 years [26,27].

## List of abbreviations used

*Kdr*: Knock-down resistance; GABA: Gamma-aminobutyric acid; LD_50_: Lethal dose 50%; GR: Granule; DT: Dispersible tablet.

## Competing interests

The authors declare that they have no competing interests.

## Authors' contributions

DF, SM, ME, AY, MMYT, VC, participated in the design of the study and writing the manuscript. PA performed the statistical analysis and writing the manuscript. All authors approved the final version of the manuscript.
